# Structural and Electrochemical Properties of Li_2_O-V_2_O_5_-B_2_O_3_-Bi_2_O_3_ Glass and Glass-Ceramic Cathodes for Lithium-Ion Batteries

**DOI:** 10.3390/molecules28010229

**Published:** 2022-12-27

**Authors:** Yuan Chen, Yufei Zhao, Feihong Liu, Mengdie Ding, Juan Wang, Jiuxin Jiang, Pascal Boulet, Marie-Christine Record

**Affiliations:** 1 Hubei Provincial Key Laboratory of Green Materials for Light Industry, Collaborative Innovation Center of Green Light-Weight Materials and Processing, School of Materials and Chemical Engineering, Hubei University of Technology, Wuhan 430068, China; 2 New Materials and Green Manufacturing Talent Introduction and Innovation Demonstration Base, Wuhan 430068, China; 3 Hubei Longzhong Laboratory, Xiangyang 441000, China; 4 MADIREL, CNRS, Faculty of Sciences, Aix-Marseille University, CEDEX 20, 13397 Marseille, France; 5 IM2NP, CNRS, Faculty of Sciences, Aix-Marseille University, CEDEX 20, 13397 Marseille, France

**Keywords:** lithium-ion battery, cathode materials, vanadate glass, structural stability, charge/discharge performance

## Abstract

In this study, 20Li_2_O-60V_2_O_5_-(20 − *x*)B_2_O_3_-*x*Bi_2_O_3_ (*x* = 5, 7.5, 10 mol%) glass materials have been prepared by the melt-quenching method, and the structure and morphology of the glass materials have been characterized by XRD, FTIR, Raman, and FE-SEM. The results show that the disordered network of the glass is mainly composed of structural motifs, such as VO_4_, BO_3_, BiO_3,_ and BiO_6_. The electrochemical properties of the glass cathode material have been investigated by the galvanostatic charge-discharge method and cyclic voltammetry, and the results show that with the increases of Bi_2_O_3_ molar content, the amount of the VO_4_ group increases, and the network structure of the glass becomes more stable. To further enhance the electrochemical properties, glass-ceramic materials have been obtained by heat treatment, and the effect of the heat treatment temperature on the structure and electrochemical properties of the glass has been studied. The results show that the initial discharge capacity of the glass-ceramic cathode obtained by heat treatment at 280 °C at a current density of 50 mA·g^−1^ is 333.4 mAh·g^−1^. In addition, after several cycles of charging and discharging at a high current density of 1000 mA·g^−1^ and then 10 cycles at 50 mA·g^−1^, its discharge capacity remains at approximately 300 mAh·g^−1^ with a capacity retention rate of approximately 90.0%. The results indicate that a proper heat treatment temperature is crucial to improving the electrochemical properties of glass materials. This study provides an approach for the development of new glass cathode materials for lithium-ion batteries.

## 1. Introduction

At present, all-solid-state lithium-ion batteries represent an important direction of development in the field of energy storage, due to their high energy density and high level of safety, and the development of solid-state lithium-ion batteries has increased the requirements for the performance of battery cathode materials [1,2,3,4]. Common lithium-ion battery cathode materials include LiFeO_4_, Li[Ni*_x_*Co*_y_*Mn*_z_*]O_2_/Li[Ni*_x_*Co*_y_*Al*_z_*]O_2_ [2,5], etc. The former has a long cycle life but a low specific capacity [6]; the latter has a high specific capacity but comes at an excessive cost. The former has a long cycle life but a low specific capacity, while the latter has a high specific capacity but a high cost [2,7]. In addition, the presence of grain boundaries reduces the efficiency of lithium-ion transport. Compared with crystalline materials, glass materials have the following advantages: (i) no grain boundaries, which is beneficial for lithium-ion transport [8]; (ii) controllable specific capacity, which can be achieved by regulating the glass component [9]; (iii) with a softening point, the softened glass is beneficial for improving the interfacial contact between the electrode material and the solid electrolyte, which further improves the lithium-ion transport efficiency [10].

Among the many glass cathode materials, vanadate glasses have been widely studied because of the multiple oxidation states of vanadium elemental [11]. Transition metal vanadium oxides are not only electrochemically active but also exhibit interesting properties [12,13,14]. For example, the conductivity of binary glass system can be improved by adding V_2_O_5_ [15,16]. However, due to the Jahn-Teller effect, vanadate glasses become structurally unstable during charging and discharging, leading to a decrease in capacity and a reduction in life cycle [17,18,19]. In order to increase the specific capacity and improve the cycling stability of vanadate glasses, several promising vanadate glass lithium-ion battery cathode materials have been reported on by researchers in recent years.

Table 1 lists some of the reported vanadate cathode materials and their cyclic properties. Du et al. [20] prepared V_2_O_5_-P_2_O_5_ glass material under a reducing atmosphere with a specific capacity of 270 mAh·g^−1^, and the capacity retention rate reached 90% after 100 cycles. Kindle et al. [21] found that the addition of Al_2_O_3_ to the V_2_O_5_-LiBO_2_ glass can stabilize the glass structure. They subsequently reported that V_2_O_5_-LiBO_2_-Al_2_O_3_ glass has good cycle stability as a cathode material [18], and its electrochemical properties can be further enhanced by ball milling, carbon coating and heat treatment. Wang et al. [22] reported for the first time the cycling stability of the Li_2_O-V_2_O_5_-B_2_O_3_ glass cathode with a high vanadium content at high current densities, and significantly enhanced its specific capacity by doping with Fe_2_O_3_.

In the above studies, the methods for improving the electrochemical performance of vanadate glass cathodes are as follows: (i) improving the preparation process [20]; (ii) carbon coating [11,18]; (iii) doping with metal oxides [18,22]; (iv) heat treatment to induce the formation of nano crystallites [18]. Bi_2_O_3_ is a semiconductor oxide with abundant resources, and it is almost non-polluting to the environment [23]. In vanadate glass systems, the addition of Bi_2_O_3_ can both change the glass network structure and improve the electrical conductivity [24], but little research has been reported on the Li_2_O-V_2_O_5_-B_2_O_3_-Bi_2_O_3_ glass system as a cathode material for lithium-ion batteries. Based on the above considerations, in this study, Li_2_O-V_2_O_5_-B_2_O_3_-Bi_2_O_3_ quaternary glass cathode materials were prepared by the melt-quenching method, and the effects of the Bi_2_O_3_ content on their structure and charge-discharge cycle stability were investigated. To further optimize the performance of the glass cathode, Li_2_O-V_2_O_5_-B_2_O_3_-Bi_2_O_3_ glass-ceramic materials were obtained by heat treatment at various temperatures, their structural and electrochemical properties were characterized, and the effects of the heat treatment temperature on their structure, specific capacity and charge/discharge cycle stability were investigated. This study provides an approach for the development of new glass cathode materials for lithium-ion batteries.

## 2. Results and Discussion

### 2.1. Structural and Morphological Characterization of LVBB Glass

**Figure 1:**
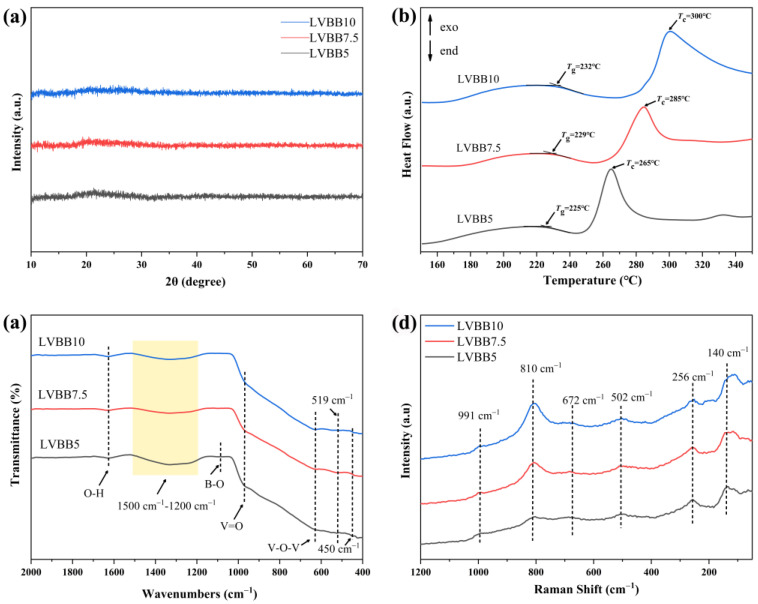
(**a**) XRD patterns, (**b**) DSC curves, (**c**) infrared spectra and (**d**) Raman spectra of LVBB5, LVBB7.5 and LVBB10 glass cathode powders. The glass was prepared by the melt-quenching method and hand-ground in an agate mortar prior to characterization.

Figure 1a shows the X-ray diffraction patterns of LVBB powders. It can be found that the three diffraction patterns have no obvious characteristic peaks and contain only gentle broad bumps, which proves that the prepared LVBB5, LVBB7.5, and LVBB10 are all glass materials. It is worth pointing out that the wide bumps in the 2*θ* range of 15–30° in Figure 1a is not obvious enough, probably because of the low short-range ordering within the quaternary glass system we studied. Figure 1b shows the DSC curves of LVBB glass powders, and one can see that the glass crystallization temperature *T*_c_ of LVBB glass increases with increasing molar content of Bi_2_O_3_; in addition, the differences between the crystallization temperature and glass transition temperature (*T*_c_–*T*_g_) of LVBB5, LVBB7.5, and LVBB10 glasses are 40 °C, 56 °C, and 68 °C, respectively. The increase in the differences (*T*_c_–*T*_g_) indicates a lower tendency towards glass crystallization and a better thermal stability of the material [25]. From Figure 1b, one can find that the glass transition temperature *T*_g_ increases slightly with the increase in Bi_2_O_3_ molar content, which may be caused by the involvement of partial Bi_2_O_3_ in the formation of the glass network structure and a reduction in the non-bridging oxygen species (NBOs) amount.

The infrared spectra of LVBB5, LVBB7.5, and LVBB10 in the range of 2000 cm^−1^–400 cm^−1^ are shown in Figure 1c, where the band near 1626 cm^−1^ is associated with the O-H bond vibration of free water in the samples [11]. The bands at 1500–1200 cm^−1^ and near 1086 cm^−1^ are attributed to asymmetric stretching vibration of the B-O bond [26,27] and symmetric stretching vibration [28,29], respectively, suggesting the presence of BO_3_ and BO_4_ groups in LVBB glass. The bands near 975 cm^−1^ and 627 cm^−1^ are related to the stretching vibrations of the V=O double bond and the V-O-V bond, respectively [11,30], suggesting the presence of a the VO_4_ group; the band near 450 cm^−1^ may come from the angular deformation vibration of the V-O bond and the vibration of the Bi-O bond in the deformed BiO_6_ group [31]. The band near 519 cm^−1^ may be related to the vibration of the Li-O bond and the vibration of the Bi-O bond in the BiO_6_ group [31,32], and the above two bands suggest the presence of the BiO_6_ group.

The Raman spectra of LVBB5, LVBB7.5, and LVBB10 in the range of 50–1200 cm^−1^ are shown in Figure 1d. The vibration peak at 991 cm^−1^ in the figure corresponds to the stretching vibration of the V=O double bond [33], and the peaks at 810 cm^−1^ and 672 cm^−1^ are attributed to the symmetric and asymmetric V-O bond stretching modes, respectively [34]. The presence of all three Raman peaks mentioned above is associated with the presence of the VO_4_ group. As shown in Figure 1d, the intensity of the peak at 810 cm^−1^ increases with the increasing molar content of Bi_2_O_3_, which indicates the increased content of VO_4_ groups in LVBB glass. More VO_4_ groups are connected to other polyhedra in the system, which can make the glass network structure more stable [18]. The peaks at 502 cm^−1^ and 256 cm^−1^ are attributed to the vibrations of B-O and V-O bonds, respectively [18], suggesting the presence of BO_3_ and VO_4_ groups. The spectral band near 140 cm^−1^ is associated with the presence of BiO_3_ and BiO_6_ groups [35], The intensity of this spectral band increases slightly with the increasing molar content of Bi_2_O_3_ as seen in Figure 1d, indicating an increase in the number of BiO_3_ and BiO_6_ groups in the glass system [36]. The structure of LVBB glass was resolved by FTIR and Raman tests, i.e., the disordered network structure of LVBB glass material is mainly composed of VO_4_, BO_3_, BO_4_, BiO_3_ and BiO_6_ structural motifs.

**Figure 2:**
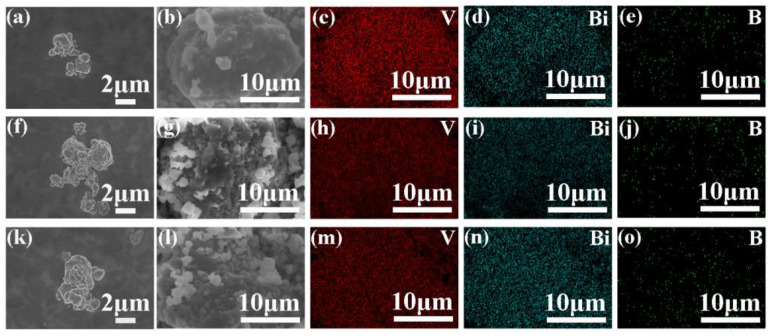
FE-SEM images of LVBB: (**a**,**b**) LVBB5 (**f**,**g**) LVBB7.5 (**k**,**i**) LVBB10; EDS spectrums of V, Bi and B element distribution in the field of view area of corresponding FE-SEM images: (**b–e**) LVBB5, (**g–j**) LVBB7.5, (**l–o**) LVBB10. The LVBB glass was ball-milled for 5 h prior to FE-SEM observation.

The microscopic morphology and surface element distribution of LVBB5, LVBB7.5, and LVBB10 glass materials after 5 h of ball milling are shown in Figure 2. Figure 2a,b,f,g,k,l show that the glass particles after ball-milling are irregular in shape; however, after ball-milling, the sharp edges of the glass powder are smoothed out. The glass particles are about 1 μm in diameter but are heavily agglomerated due to the ball-milling over a lengthy period. Figure 2c–e,h–j,m–o show the distribution of V, Bi, and B elements in the three glass materials. No significant component bias aggregation is seen in the figures, which indicates that V, Bi, and B elements are uniformly distributed in the prepared LVBB glass materials.

### 2.2. Electrochemical Properties of LVBB Glass Cathode

**Figure 3:**
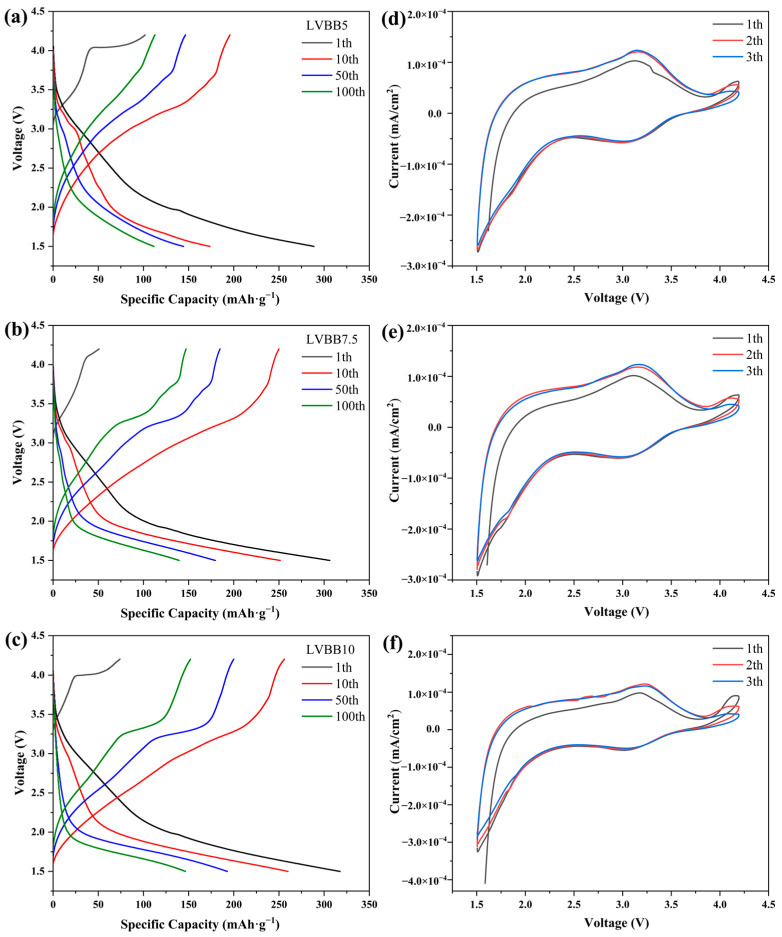
Charge and discharge curves at 50 mA·g^−1^ of (**a**) LVBB5, (**b**) LVBB7.5 and (**c**) LVBB10 in the voltage range of 1.5–4.2 V; CV curves of (**d**) LVBB5, (**e**) LVBB7.5 and (**f**) LVBB10 in the potential window of 1.5–4.2 V with a scanning rate of 0.2 mV·s^−1^.

The charge and discharge curves of LVBB5, LVBB7.5, and LVBB10 in the voltage range of 1.5–4.2 V at 50 mA·g^−1^ are shown in Figure 3a–c. It can be seen that the capacities of LVBB5, LVBB7.5, and LVBB10 samples at the first charge are 101.9, 51.2, and 74.2 mAh·g^−1^, respectively, all of which are low, indicating that fewer lithium ions can be de-intercalated in the glass structure [18]; furthermore, the appearance of a voltage plateau during the first charge may be related to the formation of the SEI film and the side reactions at the interface [37]. For both LVBB7.5 and LVBB10 glasses, voltage plateaus are observed during the 50th turn of charging, which may be related to the formation of microcrystals during the charge/discharge cycle [37]. The discharge capacities of LVBB5, LVBB7.5, and LVBB10 are 288.6, 306.2, and 317.6 mAh·g^−1^ on the first cycle, respectively, and 111.6, 139.5, and 156.4 mAh·g^−1^ on the 100th cycle, respectively, with capacity retention rates of 38.7%, 45.6%, and 49.2%, respectively.

The larger discharge capacity of LVBB10 is related to the existence of more VO_4_ groups in LVBB10. The variation in the intensity of the characteristic peaks in the Raman spectra is corroborated by the results of the electrochemical performance of the materials. As shown in Figure 1d, the intensity of the peak at 810 cm^−1^ increases with the increasing molar content of Bi_2_O_3_, which indicates the increased content of VO_4_ groups in LVBB10 glass. When the VO_4_ group is used as the main network structural unit of the glass material, there are more effective sites in its network structure, which can provide more space for lithium storage [20,22]. In addition, LVBB10 has the highest capacity retention rate, which shows that its charge/discharge cycle stability is better than that of LVBB5 and LVBB7.5. The better cyclic stability is closely related to the stability of the structure. In LVBB10, more VO_4_ groups are connected to other polyhedra in the system, which can make the glass network structure more stable. The Jahn-Teller effect has been identified in the literature as the main cause of structural instability of vanadate electrode materials, especially in liquid electrolytes where vanadium elements are continuously dissolved [18,19]. Therefore, a more stable glass structure would benefit the cycling stability of the electrode material during charge and discharge [22]. It should be noted that the dramatic decay in discharge capacities of the electrode materials after 100 cycles is also caused by the Jahn-Teller effect. In LVBB5, due to the polarization of Bi^3+^, the surrounding O^2−^ ions are attracted and the glass structure is not stable enough [36], so the discharge capacity decays too fast with cyclic charging and discharging, i.e., the cyclic stability is poor. In LVBB10, the higher the Bi_2_O_3_ content, according to the results of the previous Raman spectroscopy analysis, the more its VO_4_ group content increases, and more VO_4_ groups are connected to the bismuth-oxygen polyhedra, making the glass structure relatively stable and the cyclic stability improves.

Figure 3d–f shows the CV curves of LVBB5, LVBB7.5, and LVBB10 in the potential window of 1.5 V–4.2 V with a scanning rate of 0.2 mV·s^−1^. It can be seen from the figures that all three samples contain a pair of redox peaks, which correspond to the redox reaction of vanadium ions. The redox peaks of the first circle CV curves in all three samples are broad and weak, suggesting decomposition of the electrolyte and the formation of SEI film, which consumes a large amount of lithium ions; the formation of this SEI film leads to an irreversible capacity loss [38].

**Figure 4:**
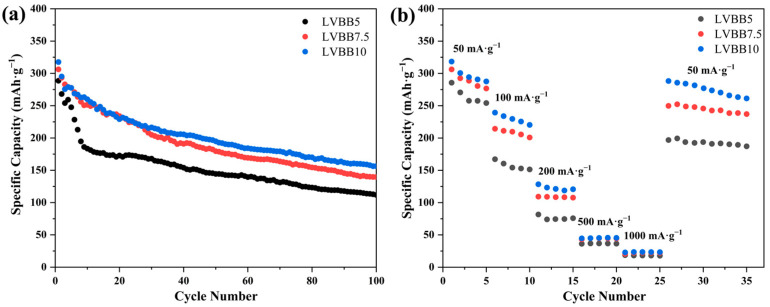
(**a**) Cycling data showing the initial 100 cycles (at 50 mA·g^−1^, in the voltage range of 1.5–4.2 V) and (**b**) rate capability tests (at different rates from 50 to 1000 mA·g^−1^) of all three samples LVBB5, LVBB7.5 and LVBB10.

Figure 4a,b show the charge/discharge cycle performance of LVBB5, LVBB7.5, and LVBB10 at 50 mA·g^−1^ and the rate capability at different current densities (at different rates from 50 to 1000 mA·g^−1^), respectively. From Figure 4a, it can be seen that the discharge capacity of LVBB5 decays rapidly during the first 10 charge/discharge cycles, from the initial 288.6 mAh·g^−1^ to 268.1 mAh·g^−1^ in the second cycle, and by the 10th cycle the capacity is only 173.7 mAh·g^−1^, with a capacity retention rate of 60.19%. Combined with the results of the FTIR, Raman, and CV analyses, we speculate that the reason for the decreasing capacity of LVBB5 is its unstable structure and the fast dissolution of vanadium in the electrolyte, which leads to the rapid decay of capacity. After the formation of SEI film, the dissolution of vanadium slows down, so the decay of discharge capacity tends to level off after 10 cycles. The results in Figure 4b show that LVBB10 keeps its discharge capacity near 275 mAh·g^−1^ after high current density (1000 mA·g^−1^) charge/discharge cycles, followed by 10 cycles at a current density of 50 mA·g^−1^, with a capacity retention rate of more than 82%, indicating that LVBB10 has a relatively optimal rate capability. Based on the above study, a stable glass network structure is beneficial to the charge/discharge cycle stability and rate capability. In order to further improve the performance of LVBB cathode material, we further heat-treated the LVBB10 and conducted a preliminary study on its structure and electrochemical properties.

### 2.3. Structural and Morphological Characterization of Li_2_O-V_2_O_5_-B_2_O_3_-Bi_2_O_3_ Glass-Ceramic Materials

**Figure 5:**
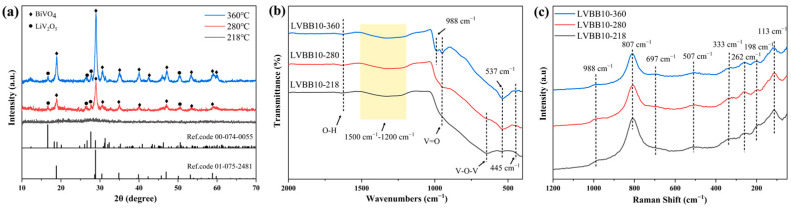
(**a**) XRD patterns, (**b**) infrared spectra and (**c**) Raman spectra of LVBB10 glass material after heat treatment at different temperatures. The ball-milled LVBB10 glass powder was heat-treated in a tube furnace with argon atmosphere for 12 h at 218 °C, 280 °C, and 360 °C, respectively.

Figure 5 shows the XRD patterns, FTIR spectra, and Raman spectra of LVBB10–218, LVBB10-280 and LVBB10-360, respectively. The results in Figure 5a show that there are no obvious diffraction peaks in the diffraction spectra of the LVBB10-218 sample, which indicates that the sample remains amorphous after heat treatment at 218 °C; LVBB10-280 and LVBB10-360 samples show obvious crystalline diffraction peaks by X-ray diffraction, and the positions of the diffraction peaks of both samples correspond to the standard spectra of BiVO_4_ (Ref. code: 98-009-6946) and LiV_2_O_5_ (Ref. code: 98-009-6946), respectively, indicating that the samples are crystallized after heat treatment. The intensity of the diffraction peaks of LVBB10-360 are stronger than those of LVBB10-280, indicating a higher crystallinity in LVBB10-360. In Figure 5b, the vibrational bands of O-H bond, B-O bond, V=O double bond, V-O-V bond, Li-O bond, and Bi-O bond of LVBB glass after heat treatment are similar to those of the unheat-treated glass samples (see Figure 1c). The differences are (i) the relatively weak absorption band of LVBB10-360 at 988 cm^−1^, which may be related to the vibration of the Bi-O bond in the BiO_3_ group [39]; (ii) the complete disappearance of the vibrational band of the V-O-V bond near 627 cm^−1^, indicating a decrease in the content of the VO_4_ group; in addition, according to the results of the previous Raman spectroscopy analysis, the reduction of VO_4_ groups is not beneficial to the stability of the glass structure, therefore, the network structure of LVBB10-360 may not be stable enough compared to the LVBB glass; (iii) compared to LVBB10-218, LVBB10-280 and LVBB10-360 show a 537 cm^−1^ absorption band, indicating an increase in the number of BiO_6_ groups, which may be related to the formation of BiVO_4_ crystals [40]. The peaks near 988, 807, 697, 507, and 262 cm^−1^ in Figure 5c are similar to those of the unheated LVBB glass samples (see Figure 1d), and for the LVBB10-280 and LVBB10-360 samples, the peaks that appear at 333, 198 and 113 cm^−1^ may be related to BiVO_4_ crystals [40]. For the three heat-treated samples, the intensity of the peaks at 807 cm^−1^ decreases with increasing heat treatment temperature, indicating a decrease in the amount of VO_4_ groups, which is consistent with the results of FTIR analysis.

**Figure 6:**
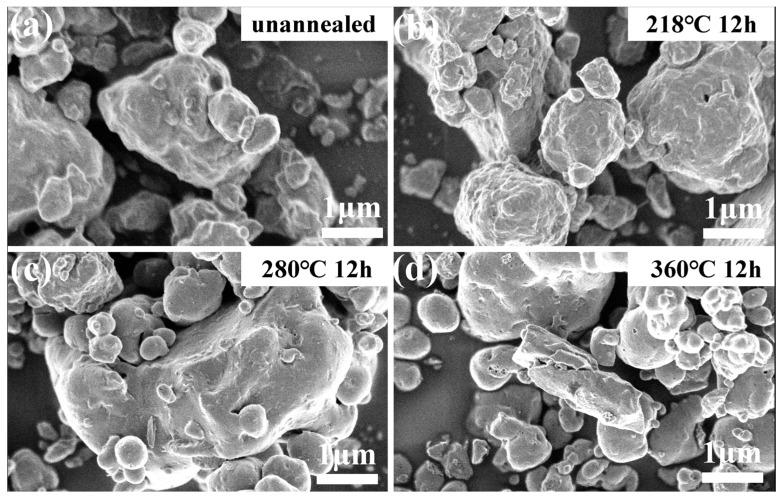
FE-SEM images of (**a**) unannealed LVBB10, (**b**) LVBB10-218, (**c**) LVBB10-280 and (**d**) LVBB10-360. The samples in (**b**–**d**) were obtained by heat-treating LVBB10 in a tube furnace with an argon atmosphere for 12 h at 218 °C, 280 °C, and 360 °C, respectively.

The FE-SEM images of LVBB10, VBB10-218, LVBB10-280, and LVBB10-360 are shown in Figure 6. Figure 6a,b show that the LVBB10 and VBB10-218 particles have irregular shapes and relatively rough surfaces; when the heat treatment temperature is further increased, the surfaces of LVBB10-280 and LVBB10-360 particles shown in Figure 6c,d become relatively smooth, implying that the internal structure of LVBB glass may have changed after heat treatment at higher temperatures, which is consistent with the XRD results.

### 2.4. Electrochemical Properties of Li_2_O-V_2_O_5_-B_2_O_3_-Bi_2_O_3_ Glass-Ceramic Cathode

**Figure 7:**
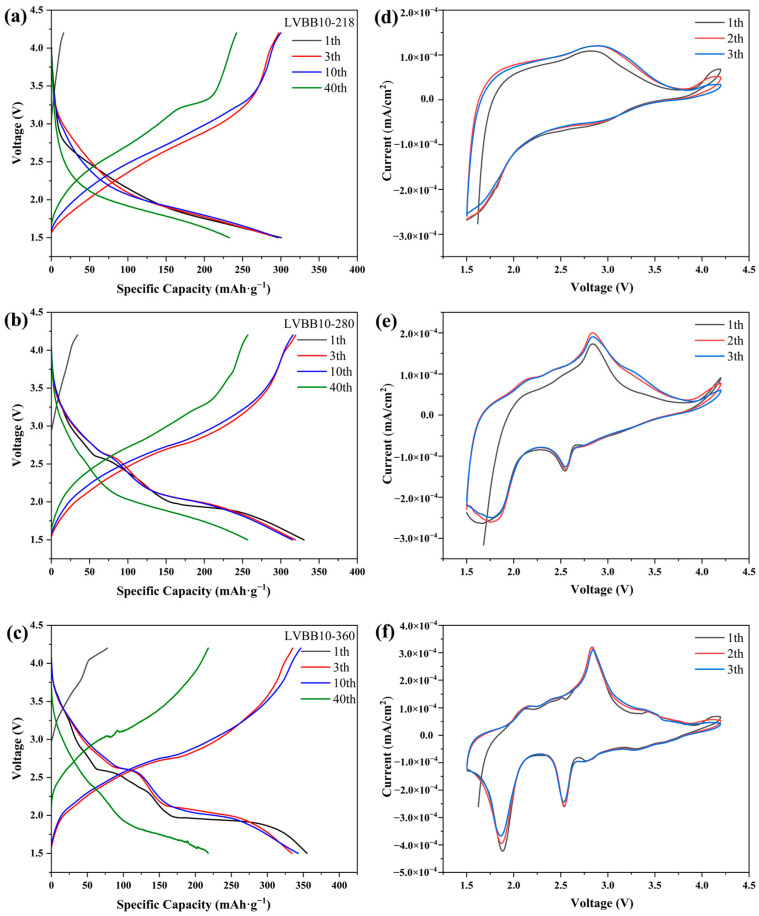
Charge and discharge curves at 50 mA·g^−1^ of (**a**) LVBB-218, (**b**) LVBB-280 and (**c**) LVBB-360 in the voltage range of 1.5–4.2 V; CV curves of (**d**) LVBB-218, (**e**) LVBB-280 and (**f**) LVBB-360 in the potential window of 1.5 V–4.2 V with a scanning rate of 0.2 mV·s^−1^.

The charge and discharge curves of LVBB10-218, LVBB10-280, and LVBB10-360 in the voltage range of 1.5–4.2V at 50 mA·g^−1^ are shown in Figure 7a–c, and the results show that the capacities of the LVBB10-218, LVBB10-280, and LVBB10-360 samples at the first discharge are 300.6, 333.4 and 355.2 mAh·g^−1^, and the discharge capacities after 40 cycles are 232.6, 256.2, and 217.63 mAh·g^−1^, with capacity retention rates of 77.4%, 76.8%, and 61.3%, respectively. LVBB10-360 has the highest initial discharge capacity and LVBB10-218 has the highest capacity retention, suggesting that the heat treatment has a positive effect on the electrochemical properties of LVBB glass, probably due to the heat treatments further stabilizing the material structure and removing some of the internal stresses in the glass [18,21]. The LVBB10-280 and LVBB10-360 samples show a discharge voltage plateau during the first 10 cycles of discharge, while the plateau has disappeared by the 40th charge/discharge cycle, suggesting a decrease in crystallinity [18]. LVBB10-218 remains amorphous after the heat treatment, so there is no voltage plateau, and only a gentler slope. Figure 7d–f show the CV curves of LVBB10-218, LVBB10-280, and LVBB10-360 in the potential window of 1.5 V–4.2 V with a scanning rate of 0.2 mV·s^−1^. The pair of broad redox peaks in Figure 7d, corresponding to the redox process of vanadium ions, can be observed as the potential difference Δ*E*_p_ between the oxidation and reduction peaks, which is smaller than that of the unannealed glass (see Figure 3f), suggesting a better intercalation/de-intercalation reversibility of lithium ions [41]. The two pairs of redox peaks at 2.55/2.84 V and 2.76/2.43 V in Figure 7e are associated with V^3+^/V^4+^ and V^4+^/V^5+^ redox reactions [22]. The number of redox peaks of LVBB10-360 in Figure 7f is higher than in the rest of the samples, and the unpaired appearance of redox peaks may be related to the generation of irreversible phases in the glass [42]; these resulting crystals due to heat treatment may be involved in the intercalation/de-intercalation process of lithium ions.

**Figure 8:**
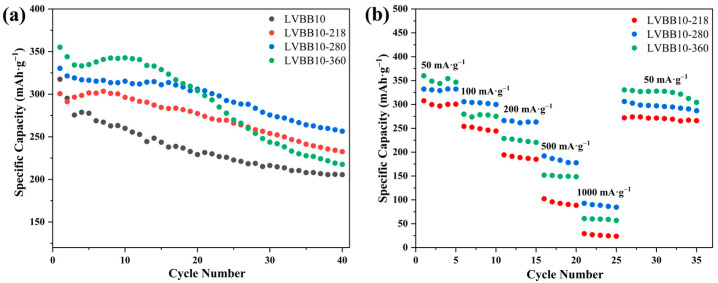
(**a**) Cycling data showing the initial 40 cycles (at 50 mA·g^−1^, in the voltage range of 1.5–4.2 V) of LVBB10, LVBB10-218, LVBB10-280 and LVBB10-360; (**b**) rate capability tests (at different rates from 50 to 1000 mA·g^−1^) of LVBB10-218, LVBB10-280 and LVBB10-360 samples.

Figure 8a,b show the charge/discharge cycle performance of the LVBB10-218, LVBB10-280, and LVBB10-360 samples at 50 mA·g^−1^ and the rate capability at different current densities (at different rates from 50 to 1000 mA·g^−1^), respectively. The results in Figure 8a show that LVBB10-360 has the highest initial specific capacity of 355.2 mAh·g^−1^ compared to the other samples, which is related to the irreversible phase generated in the glass matrix [42]. The irreversible phase (LiV_2_O_5_) provides additional capacity in the initial few cycles, hence the initial capacity is high, and a clear voltage plateau appears in the discharge curve (see Figure 7c); however, as the intercalation/de-intercalation process of lithium ions proceeds, the crystal structure of LiV_2_O_5_ is disrupted, the discharge voltage plateau gradually disappears (see Figure 7c), and the capacity also decreases rapidly in the first three cycles. The discharge capacity of LVBB10-360 decays too fast, indicating its poor charge/discharge cycle stability, which is due to the higher crystallinity of LVBB10-360 compared to LVBB10, LVBB10-218 and LVBB10-280. The higher crystallinity means that LVBB10-360 is not as structurally loose as the other samples and is more affected by the volume changes caused by the intercalation/de-intercalation of lithium ions. After 40 cycles of charging and discharging, the discharge capacity retention rates of LVBB10-218 and LVBB10 are 77.4% and 64.3%, respectively, which shows that LVBB10-218 possesses higher cycling stability than LVBB10, which may be related to the heat treatment that eliminates some internal stresses inside the glass [18]. In Figure 8b, LVBB10-280 is shown to be able to maintain a discharge capacity of 84.7~93.2 mAh·g^−1^ at a current density of 1000 mA·g^−1^, which is higher than that of the rest of the samples. In addition, after high current density cycles and then 10 cycles of charging and discharging at a current density of 50 mA·g^−1^, its discharge capacity remains at approximately 300 mAh·g^−1^ with a capacity retention rate of approximately 90.0%, indicating that among all samples, the LVBB10-280 has the best performance of rate capability.

In summary, it is not difficult to outline the principle of the influence of heat treatment temperature on the electrochemical properties of LVBB glass: A sufficiently high heat treatment temperature can induce the crystallization of LVBB glass, and the new phase formed can provide additional specific capacity to the electrode material; but when the heat treatment temperature is too high, the excessive crystallinity will in turn make the structure of the material unstable and lead to a decrease in charge/discharge stability. Therefore, it is crucial to select the appropriate heat treatment temperature to improve the electrochemical properties of LVBB glass.

## 3. Materials and Methods

### 3.1. Preparation of Glass Electrode Materials

Lithium hydroxide monohydrate (LiOH·H_2_O), vanadium pentoxide (V_2_O_5_), boron oxide (B_2_O_3_) and bismuth oxide (Bi_2_O_3_) were used as raw materials (all purchased from Shanghai Macklin Biochemical Co., China, with a purity of AR grade or above) to prepare by the melt-quenching method, the 20Li_2_O-60V_2_O_5_-(20 − *x*)B_2_O_3_-*x*Bi_2_O_3_ (*x* = 5, 7.5, 10, molar percent) glass samples that are hereafter named LVBB5, LVBB7.5, and LVBB10, respectively. The raw materials were weighed according to the stoichiometric ratio, mixed uniformly, then placed in a corundum crucible, melted at 900 °C in an air atmosphere, held for 2 h, then quenched between two copper plates to obtain flake glass samples. The flake glass samples were grounded in an agate mortar, and the ground glass was then added to *n*-hexane and ball-milled in a planetary ball mill at a speed of 400 rpm for 5 h. Finally, the *n*-hexane was fully removed by baking at 125 °C to obtain the glass electrode materials. The LVBB10 glass powder after ball milling was heat-treated in a tube furnace with an argon atmosphere at 218 °C, 280 °C, and 360 °C for 12 h. The heat-treated samples were named LVBB10-218, LVBB10-280, and LVBB10-360, respectively.

### 3.2. Characterization of the Glass Materials

The phase analysis of the samples were characterized by X-ray diffraction (XRD) using an X-ray diffractometer (PANalytical, EMPYREAN) with Cu K*α* radiation (*λ* = 0.154 nm) and a 2*θ* scan range of 10–70° at a rate of 2°/min. Differential scanning calorimeter (DSC, Q600, NETZSCH) was used at a heating rate of 10 °C/min to measure the glass transition temperature (*T*_g_) and the crystallization temperature (*T*_c_) of the glass powders. The samples were structurally characterized by Fourier transform infrared spectroscopy (FTIR, Tensor27, BRUKER) in the range 400–2000 cm^−1^, and by Raman microscopy (Raman, XploRA PLUS, HORIBA) in the range 50–1200 cm^−1^, for which the wavelength of the laser source was 532 nm. Prior to Raman testing, all powder samples were sieved and then spread on the conductive adhesive uniformly. In addition, the excitation spot size, laser power, and exposure time were consistent from one test to the next. The morphology and elements distribution of the samples have been determined by a field-emission scanning electron microscope (FE-SEM, SU8010, HITACHI) operating at 5 kV with an energy dispersive X-ray spectrometer (EDS, X-Max^N^, OXFORD), respectively.

### 3.3. Electrochemical Tests

The glass cathode material, cotinine black (Shanghai Macklin Biochemical Co., Shanghai, China, AR) and the polyvinylidene fluoride (PVDF, Shanghai Macklin Biochemical Co., Shanghai, China, AR) were weighed according to the mass ratio of 7:2:1, mixed well, and the mixed sample was then placed in a beaker with N-Methylpyrrolidone (NMP, Shanghai Aladdin Biochemical Co., Shanghai, China, AR) to be magnetically stirred for 7 h. The slurry was coated on 20 μm thick aluminum foil, fully dried at 80 °C, and finally cut into 15 mm diameter discs. 1 M LiPF6 dissolved in ethylene carbonate (EC) and dimethyl carbonate (DMC) as electrolyte (EC:DMC = 1:1 vol%). Polypropylene film (Celgard 2500, Celgard Inc., Charlotte, NC, USA) was used as a separator, and CR2032 half-cells were assembled in a glove box in an argon atmosphere. A Land CT2001A battery test system was used to measure the charge/discharge cycling performance and rate capability of the cathode material in the voltage range of 1.5–4.2 V (vs. Li/Li^+^). Cyclic voltammetry (CV) analysis of the cathode material was performed using an electrochemical workstation (CS350M, CORRTEST) with a voltage window of 1.5–4.2 V and a scan rate of 0.2 mV·s^−1^.

## 4. Conclusions

In this study, 20Li_2_O-60V_2_O_5_-(20 − *x*)B_2_O_3_-*x*Bi_2_O_3_ (*x* = 5, 7.5, 10 mol%) glass materials were prepared by the melt-quenching method. The disordered network of the glass is mainly composed of structural motifs such as VO_4_, BO_3_, BiO_3,_ and BiO_6_, and with the increases in Bi_2_O_3_ molar content, the amount of the VO_4_ group increases, and the network structure of the glass becomes more stable. The glass cathode with 10 mol% Bi_2_O_3_ has the best charge/discharge cycle stability and rate capability, which is attributed to the increased VO_4_ group amount that makes the structure of the glass material more stable.

A heat treatment can help further enhance the charge/discharge cycle stability of the glass materials. The initial discharge capacity of the glass-ceramic cathode obtained by heat treatment at 280 °C is 333.4 mAh·g^−1^, and the discharge capacity at a current density of 1000 mA·g^−1^ maintains at 84.7 to 93.2 mAh·g^−1^. In addition, after several cycles of charging and discharging at a high current density of 1000 mA·g^−1^ followed by 10 cycles at 50 mA·g^−1^, its discharge capacity remains at approximately 300 mAh·g^−1^ with a capacity retention rate of approximately 90.0%. Choosing the proper heat treatment temperature is crucial to improve the electrochemical properties of glass materials. Too low a heat treatment temperature (below *T*_g_) can result in insufficient new phases being formed in the glass to provide additional specific capacity, while too high a heat treatment temperature can lead to structural instability and thus a decrease in charge/discharge stability.

## Figures and Tables

**Table 1:** Different vanadate cathode materials and their cycling properties reported in the literatures.

Cathode Material	Cycle Number	Current Density (mA·g^−1^)	Specific Capacity (mAh·g^−1^)	Capacity Retention	Reference
Crystalline Zn_0.29_V_2_O_5_	100	63.2	316	104	[13]
Crystalline Mn_2_V_2_O_7_	1142	344	242	82	[14]
Glass V_2_O_5_-P_2_O_5_	100	17	270	90	[20]
Glass V_2_O_5_-LiBO_2_	10	50	327	88	[21]
Glass RGO/V_2_O_5_-LiBO_2_	100	50	400	75	[21]
Glass Al_2_O_3_-V_2_O_5_-LiBO_2_	100	50	250 (after rate capability testing)	50	[18]
Glass Li_2_O-V_2_O_5_-B_2_O_3_	100	100	170	81.7	[22]
Glass Li_2_O-V_2_O_5_-B_2_O_3_-Fe_2_O_3_	100	100	306.2	39.3	[22]
Glass Li_2_O-V_2_O_5_-B_2_O_3_-Bi_2_O_3_	100	50	317.6	49.2	Present work

## Data Availability

Not applicable.

## References

[1] Bates A.M., Preger Y., Torres-Castro L., Harrison K.L., Harris S.J., Hewson J. (2022). Are Solid-State Batteries Safer than Lithium-Ion Batteries?. Joule.

[2] Nitta N., Wu F., Lee J.T., Yushin G. (2015). Li-Ion Battery Materials: Present and Future. Mater. Today.

[3] Salgado R.M., Danzi F., Oliveira J.E., El-Azab A., Camanho P.P., Braga M.H. (2021). The Latest Trends in Electric Vehicles Batteries. Molecules.

[4] Bhat V.S., Kanagavalli P., Sriram G., B R.P., John N.S., Veerapandian M., Kurkuri M., Hegde G. (2020). Low Cost, Catalyst Free, High Performance Supercapacitors Based on Porous Nano Carbon Derived from Agriculture Waste. J. Energy Storage.

[5] Noh H.-J., Youn S., Yoon C.S., Sun Y.-K. (2013). Comparison of the Structural and Electrochemical Properties of Layered Li[Ni*_x_*Co*_y_*Mn*_z_*]O_2_ (*x* = 1/3, 0.5, 0.6, 0.7, 0.8 and 0.85) Cathode Material for Lithium-Ion Batteries. J. Power Sources.

[6] Park Y., Kim S., Jin S., Lee S., Noda I., Jung Y. (2019). Investigation of the Phase Transition Mechanism in LiFePO_4_ Cathode Using In Situ Raman Spectroscopy and 2D Correlation Spectroscopy during Initial Cycle. Molecules.

[7] Zhang C.-M., Li F., Zhu X.-Q., Yu J.-G. (2022). Triallyl Isocyanurate as an Efficient Electrolyte Additive for Layered Oxide Cathode Material-Based Lithium-Ion Batteries with Improved Stability under High-Voltage. Molecules.

[8] Uchaker E., Zheng Y.Z., Li S., Candelaria S.L., Hu S., Cao G.Z. (2014). Better than Crystalline: Amorphous Vanadium Oxide for Sodium-Ion Batteries. J. Mater. Chem. A.

[9] Lee J., Urban A., Li X., Su D., Hautier G., Ceder G. (2014). Unlocking the Potential of Cation-Disordered Oxides for Rechargeable Lithium Batteries. Science.

[10] Yamauchi H., Ikejiri J., Tsunoda K., Tanaka A., Sato F., Honma T., Komatsu T. (2020). Enhanced Rate Capabilities in a Glass-Ceramic-Derived Sodium All-Solid-State Battery. Sci. Rep..

[11] Afyon S., Krumeich F., Mensing C., Borgschulte A., Nesper R. (2015). New High Capacity Cathode Materials for Rechargeable Li-Ion Batteries: Vanadate-Borate Glasses. Sci. Rep..

[12] Vernardou D., Drosos C., Kafizas A., Pemble M.E., Koudoumas E. (2020). Towards High Performance Chemical Vapour Deposition V_2_O_5_ Cathodes for Batteries Employing Aqueous Media. Molecules.

[13] Shreenivasa L., Yogeeshwari R.T., Viswanatha R., Sriram G., Kalegowda Y., Kurkuri M.D., Ashoka S. (2019). An Introduction of New Nanostructured Zn_0.29_V_2_O_5_ Cathode Material for Lithium Ion Battery: A Detailed Studies on Synthesis, Characterization and Lithium Uptake. Mater. Res. Express.

[14] Shreenivasa L., Viswanatha R., Ganesan S., Kalegowda Y., Kurkuri M.D., Ashoka S. (2020). Scalable Chemical Approach to Prepare Crystalline Mn_2_V_2_O_7_ Nanoparticles: Introducing a New Long-Term Cycling Cathode Material for Lithium-Ion Battery. J. Mater. Sci. Mater. Electron..

[15] Lee Y. (2004). Li-Ion Conductivity in Li_2_O-B_2_O_3_-V_2_O_5_ Glass System. Solid State Ion..

[16] Jozwiak P., Garbarczyk J. (2005). Mixed Electronic–Ionic Conductivity in the Glasses of the Li_2_O-V_2_O_5_-P_2_O_5_ System. Solid State Ion..

[17] Shao Y., Lu Z., Li L., Liu Y., Yang L., Shu T., Li X., Liao S. (2022). Significant Enhancement of the Capacity and Cycling Stability of Lithium-Rich Manganese-Based Layered Cathode Materials via Molybdenum Surface Modification. Molecules.

[18] Kindle M., Cha Y., McCloy J.S., Song M.-K. (2021). Alternatives to Cobalt: Vanadate Glass and Glass-Ceramic Structures as Cathode Materials for Rechargeable Lithium-Ion Batteries. ACS Sustain. Chem. Eng..

[19] Fu X., Pu X., Wang H., Zhao D., Liu G., Zhao D., Chen Z. (2019). Understanding Capacity Fading of the LiVO_3_ Cathode Material by Limiting the Cutoff Voltage. Phys. Chem. Chem. Phys..

[20] Du M., Huang K., Guo Y., Xie Z., Jiang H., Li C., Chen Y. (2019). High Specific Capacity Lithium Ion Battery Cathode Material Prepared by Synthesizing Vanadate–Phosphate Glass in Reducing Atmosphere. J. Power Sources.

[21] Kindle M., Kmiec S., d’Anciães Almeida Silva I., Eckert H., Martin S.W., Song M.-K., McCloy J.S. (2019). Structural Properties of Alumina-Doped Lithium Borovanadate Glasses and Glass-Ceramics. J. Non-Cryst. Solids.

[22] Wang H., Li J., Yin Y., Chen J., Wang L., Zhang P., Lai X., Yue B., Hu X., He D. (2021). Effects of V_2_O_5_ and Fe_2_O_3_ on the Structures and Electrochemical Performances of Li_2_O-V_2_O_5_-B_2_O_3_ Glass Materials in Lithium-Ion Batteries. J. Alloys Compd..

[23] Kaur P., Singh K.J., Kurudirek M., Thakur S. (2019). Study of Environment Friendly Bismuth Incorporated Lithium Borate Glass System for Structural, Gamma-Ray and Fast Neutron Shielding Properties. Spectrochim. Acta Part A Mol. Biomol. Spectrosc..

[24] Shapaan M. (2017). Structure and Electric Conductivity of Mixed Electronic-Ionic Bi_2_O_3_-Li_2_O-V_2_O_5_-B_2_O_3_ Glass System. Int. J. Thin Fil. Sci. Tec..

[25] Margha F.H., El-Bassyouni G.T., Turky G.M. (2019). Enhancing the Electrical Conductivity of Vanadate Glass System (Fe_2_O_3_, B_2_O_3_, V_2_O_5_) via Doping with Sodium or Strontium Cations. Ceram. Int..

[26] Dantas N.O., Ayta W.E.F., Silva A.C.A., Cano N.F., Silva S.W., Morais P.C. (2011). Effect of Fe_2_O_3_ Concentration on the Structure of the SiO_2_-Na_2_O-Al_2_O_3_-B_2_O_3_ Glass System. Spectrochim. Acta Part A Mol. Biomol. Spectrosc..

[27] Abdel-Hameed S.A.M., Fathi A.M., Elwan R.L., Margha F.H. (2020). Effect of F^−^ and B^3+^ Ions and Heat Treatment on the Enhancement of Electrochemical and Electrical Properties of Nanosized LiTi_2_(PO_4_)_3_ Glass-Ceramic for Lithium-Ion Batteries. J. Alloys Compd..

[28] Laorodphan N., Pooddee P., Kidkhunthod P., Kunthadee P., Tapala W., Puntharod R. (2016). Boron and Pentavalent Vanadium Local Environments in Binary Vanadium Borate Glasses. J. Non-Cryst. Solids.

[29] Saetova N.S., Raskovalov A.A., Antonov B.D., Yaroslavtseva T.V., Reznitskikh O.G., Zabolotskaya E.V., Kadyrova N.I., Telyatnikova A.A. (2018). Conductivity and Spectroscopic Studies of Li_2_O-V_2_O_5_-B_2_O_3_ Glasses. Ionics.

[30] Mandal S., Hazra S., Das D., Ghosh A. (1995). Structural Studies of Binary Iron Vanadate Glass. J. Non-Cryst. Solids.

[31] Baia L., Stefan R., Kiefer W., Popp J., Simon S. (2002). Structural Investigations of Copper Doped B_2_O_3_–Bi_2_O_3_ Glasses with High Bismuth Oxide Content. J. Non-Cryst. Solids.

[32] ElBatal F.H., Abdelghany A.M., Ezz ElDin F.M., ElBatal H.A. (2020). Vanadium Structural Role in Binary Fluoride Borate Glasses and Effects of Gamma Irradiation. Radiat. Phys. Chem..

[33] Garbarczyk J. (2000). Studies of Silver-Vanadate-Phosphate Glasses by Raman, EPR and Impedance Spectroscopy Methods. Solid State Ion..

[34] Galembeck A., Alves O.L. (2000). BiVO_4_ Thin Film Preparation by Metalorganic Decomposition. Thin Solid Films.

[35] Bale S., Rao N.S., Rahman S. (2008). Spectroscopic Studies of Bi_2_O_3_-Li_2_O-ZnO-B_2_O_3_ Glasses. Solid State Sci..

[36] Hardcastle F.D., Wachs I.E., Eckert H., Jefferson D.A. (1991). Vanadium(V) Environments in Bismuth Vanadates: A Structural Investigation Using Raman Spectroscopy and Solid State 51V NMR. J. Solid State Chem..

[37] Kong F., Yi L., Huang S., Liang X., Rao Y., Su Z., Li C., Jiang H. (2021). Using Glass Defect Engineering to Obtain Order-Disorder Transformation in Cathode for High Specific Capacity Lithium Ion Battery. Appl. Surf. Sci..

[38] Esper J.D., Zhuo Y., Barr M.K.S., Yokosawa T., Spiecker E., de Ligny D., Bachmann J., Peukert W., Romeis S. (2020). Shape-Anisotropic Cobalt-Germanium-Borate Glass Flakes as Novel Li-Ion Battery Anodes. Powder Technol..

[39] Singh K. (1996). Electrical Conductivity of Li_2_O-B_2_O_3_-Bi_2_O_3_: A Mixed Conductor. Solid State Ion..

[40] Liu J., Wang H., Wang S., Yan H. (2003). Hydrothermal Preparation of BiVO_4_ Powders. Mater. Sci. Eng. B.

[41] Yang Z., Xiang W., Wu Z., He F., Zhang J., Xiao Y., Zhong B., Guo X. (2017). Effect of Niobium Doping on the Structure and Electrochemical Performance of LiNi_0.5_Co_0.2_Mn_0.3_O_2_ Cathode Materials for Lithium Ion Batteries. Ceram. Int..

[42] Liu P., Wang B., Sun X., Gentle I., Zhao X.S. (2016). A Comparative Study of V_2_O_5_ Modified with Multi-Walled Carbon Nanotubes and Poly(3,4-Ethylenedioxythiophene) for Lithium-Ion Batteries. Electrochim. Acta.

